# In Vitro Metabolism and p53 Activation of Genotoxic Chemicals: Abiotic CYP Enzyme vs Liver Microsomes

**DOI:** 10.1021/acs.chemrestox.4c00101

**Published:** 2024-06-20

**Authors:** Luise Henneberger, Julia Huchthausen, Jenny Braasch, Maria König, Beate I. Escher

**Affiliations:** † Helmholtz Centre for Environmental Research—UFZ, Department of Cell Toxicology, Permoserstr. 15, 04318 Leipzig, Germany; ‡ Eberhard Karls University Tübingen, Environmental Toxicology, Department of Geosciences, 72076 Tübingen, Germany

## Abstract

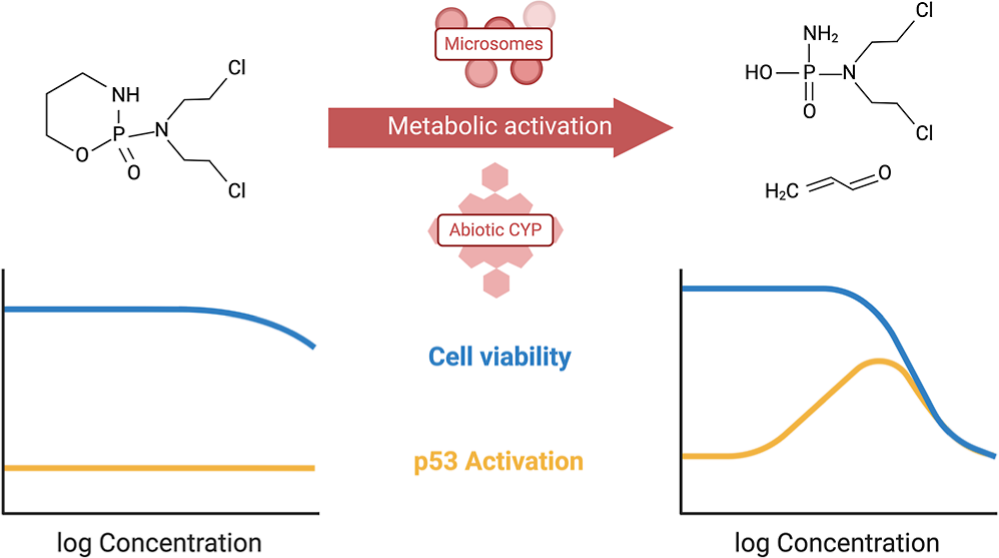

Chemicals often require metabolic activation to become genotoxic. Established test guidelines recommend the use of the rat liver S9 fraction or microsomes to introduce metabolic competence to *in vitro* cell-based bioassays, but the use of animal-derived components in cell culture raises ethical concerns and may lead to quality issues and reproducibility problems. The aim of the present study was to compare the metabolic activation of cyclophosphamide (CPA) and benzo[*a*]pyrene (BaP) by induced rat liver microsomes and an abiotic cytochrome P450 (CYP) enzyme based on a biomimetic porphyrine catalyst. For the detection of genotoxic effects, the chemicals were tested in a reporter gene assay targeting the activation of the cellular tumor protein p53. Both chemicals were metabolized by the abiotic CYP enzyme and the microsomes. CPA showed no activation of p53 and low cytotoxicity without metabolic activation, but strong activation of p53 and increased cytotoxicity upon incubation with liver microsomes or abiotic CYP enzyme. The effect concentration causing a 1.5-fold induction of p53 activation was very similar with both metabolization systems (within a factor of 1.5), indicating that genotoxic metabolites were formed at comparable concentrations. BaP also showed low cytotoxicity and no p53 activation without metabolic activation. The activation of p53 was detected for BaP upon incubation with active and inactive microsomes at similar concentrations, indicating experimental artifacts caused by the microsomes or NADPH. The activation of BaP with the abiotic CYP enzyme increased the cytotoxicity of BaP by a factor of 8, but no activation of p53 was detected. The results indicate that abiotic CYP enzymes may present an alternative to rat liver S9 fraction or microsomes for the metabolic activation of test chemicals, which are completely free of animal-derived components. However, an amendment of existing test guidelines would require testing of more chemicals and genotoxicity end points.

## Introduction

Cell-based *in vitro* bioassays are considered alternatives to animal testing for chemical hazard assessment. However, the majority of cell culture laboratories still rely on a variety of animal-derived components, including fetal bovine serum (FBS) as a nutrient supply, trypsin for cell detachment, or antibodies for staining. Test chemicals may also require metabolic activation to exhibit adverse effects, but most cell lines used in cell-based *in vitro* bioassays have no or very low basal cytochrome P450 (CYP) activity.^1−4^ Therefore, various methods for introducing metabolic capacity to bioassays have been described. The most common approaches include the use of induced rat liver S9 fraction, either directly dosed to the cells^5^ or embedded in an alginate matrix,^6^ and human or rat liver microsomes.^7^ Induced rat liver S9 fraction is also recommended for the metabolic activation of test chemicals in the OECD Test Guideline no. 487 (*In Vitro* Mammalian Cell Micronucleus Test).^8^ Despite the extensive use of rat liver S9 fraction and liver microsomes, it has to be considered that both materials show high batch to batch variability and can cause additional cytotoxicity.^9^ In addition, these materials also reduce the freely dissolved concentration of test chemicals^10^ and may interfere with cell imaging.^11^ Furthermore, employing the liver S9 fraction and liver microsomes from rodents contradicts the use of bioassays as alternatives to animal testing.

For genotoxicity testing, only Phase I metabolism is of interest (i.e., oxidation), as in most cases, Phase II metabolism leads to detoxification of the test chemicals. In general, genotoxic chemicals can be divided into two different categories, clastogens and aneugens, based on their mode of action. Clastogens are reactive chemicals that cause DNA-damage and double-strand breaks (e.g., mitomycin C or cytosine arabinoside). A subgroup of clastogens are promutagens that show no DNA reactivity themselves but are metabolized by CYP enzymes to form reactive metabolites (e.g., polycyclic aromatic hydrocarbons like benzo[*a*]pyrene (BaP)). Aneugens are not DNA-reactive but affect the spindle apparatus during cell division, leading to an abnormal number of chromosomes (e.g., colchicine or vinblastine).

**Figure 1:**
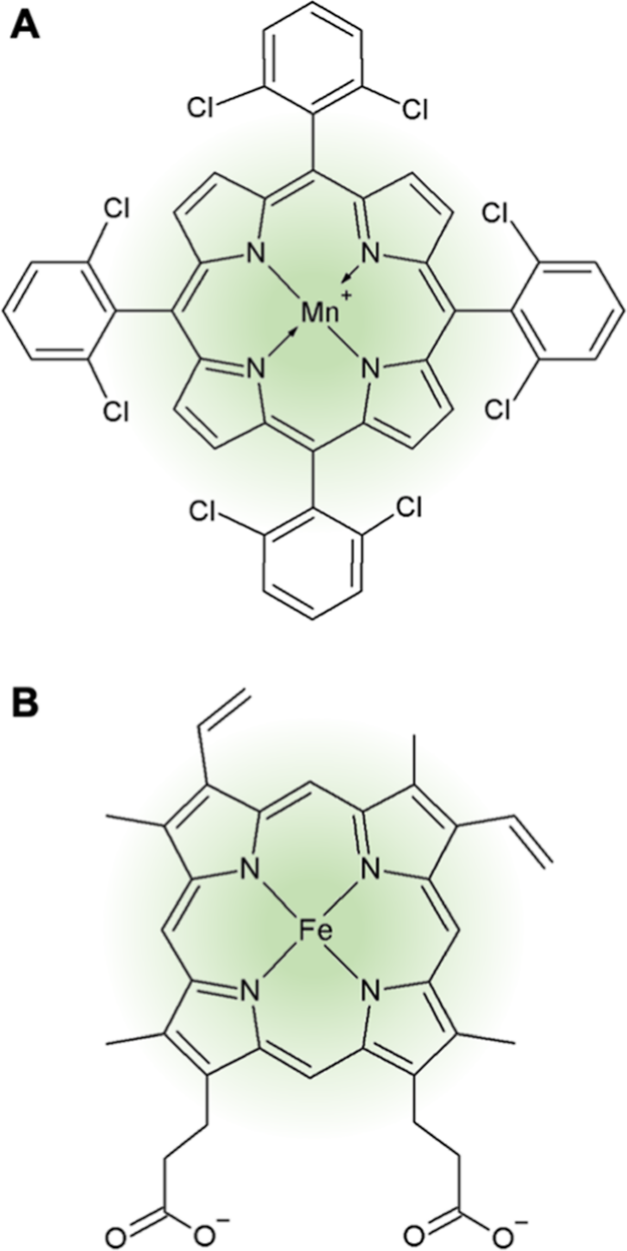
(A) Structure of biomimetic catalyst 5,10,15,20-tetrakis(2,6-dichlorophenyl)-porphyrin-Mn(III) chloride (TDCPP) and (B) prosthetic heme group of CYP monooxygenases (heme *b*) adapted from Sono et al.^12^

Already in 1979, Groves et al.^13^ described a new approach for simulating oxidation reactions by CYP enzymes utilizing so-called “biomimetic catalysts”. Many different structures have been described in the literature in the past 45 years, but the most promising catalysts belong to the group of metalloporphyrins, mostly with manganese or iron as the central ion.^14−16^ The structure of the metalloporphyrins is remarkably similar to the prosthetic heme group of CYP enzymes, explaining their shared catalytic function (Figure 1).^17,18^ Depending on the functional groups attached to the porphyrin, the catalysts have different properties. Some catalysts are soluble in water, but often the reaction can only be performed in solvents, e.g., acetonitrile, methanol, benzene, or dichloromethane.^16^ The majority of the catalysts require a single oxygen donor for the reaction (e.g., hydrogen peroxide, sodium hypochlorite, iodosylbenzene, potassium monoperoxysulfate, magnesium monoperoxyphthalate, or *tert*-butyl hydroperoxide)^16^ and only a few structures are able to directly catalyze the oxidation with elemental oxygen.^19^ Furthermore, the addition of a cocatalyst (e.g., ammonium acetate, imidazole, or ascorbate) is usually required to facilitate the reaction.^17^

A main application of biomimetic catalysts is the production of drug metabolites.^20,21^ Few studies have also applied biomimetic catalysts for the activation of test chemicals in bacterial toxicity assays (Ames Test),^22,23^ but no combination with bioassays using human cells has been reported so far. The aim of the present study was therefore to explore the use of a biomimetic catalyst (5,10,15,20-tetrakis(2,6-dichlorophenyl)-porphyrin-Mn(III) chloride, TDCPP, Figure 1A) as an abiotic CYP analogue for the metabolic activation of two genotoxic reference compounds, cyclophosphamide (CPA) and BaP,^8^ as an alternative to (induced) rat liver microsomes. Furthermore, we assessed the applicability of the abiotic CYP enzyme for a genotoxicity assay based on genetically modified HCT-116 cells using the activation of the cellular tumor protein p53 as the toxicological end point. The activation of p53 indicates DNA damage, e.g., caused by clastogenic chemicals, and leads to cell cycle arrest and apoptosis.^24^

## Materials and Methods

### Chemicals

Carbamazepine (Cayman Chemical Co.), carbamazepine 10,11-epoxide (CAS 36507-30-9, HPC Standards), CPA (CAS 50-18-0, Sigma-Aldrich), BaP (CAS 50-32-8, Sigma-Aldrich), 3-hydroxybenzo[*a*]pyrene (CAS 13345-21-6, Biozol), benzo[*a*]pyrene-7,8-dihydrodiol 9,10-epoxide (BPDE, CAS 72485–26–8, Sigma-Aldrich), and benzo[*a*]pyrene-6,12-quinone (CAS 3067-12-7, Toronto Research Chemicals) had a purity of at least 96%. Additional metabolites of BaP were acquired from the Biochemical Institute for Environmental Carcinogens: 9-hydroxybenzo[*a*]pyrene (CAS 17573-21-6), (±)-trans-4,5-dihydroxy-4,5-dihydrobenzo[*a*]pyrene (CAS 37571-88-3), (±)-trans-7,8-dihydroxy-7,8-dihydrobenzo[*a*]pyrene (CAS 60864-95-1), and (±)-trans-9,10-dihydroxy-9,10-dihydrobenzo[*a*]pyrene (CAS 58886-98-9). The biomimetic catalyst 5,10,15,20-tetrakis(2,6-dichlorophenyl)-porphyrin-Mn(III) chloride (TDCPP) was purchased from abcr. Acetonitrile (UHPLC-MS grade) and methanol (for LC–MS) from Chemsolute and dichloromethane (Lichrosolv) from Merck were used. Water was purified by using a Milli-Q water purification system from Merck. Ammonium acetate from Honeywell, formic acid from Serva, hydrogen peroxide (30% aqueous solution) from Sigma-Aldrich, and β-nicotine amide adenine dinucleotide phosphate (NADPH) tetrasodium salt (≥95%) from Carl Roth were used. Catalase from *Micrococcus lysodeikticus* was acquired from Sigma-Aldrich (cat. no. 60634). The activity of the catalase batch used for the present study was 123,945 U/mL. Liver microsomes (20 mg/mL) from male Sprague–Dawley rats treated with β-naphthoflavone and phenobarbital were purchased from Xenotech (cat. no. R1081).

### Protocol for Oxidation of Test Chemicals by Abiotic CYP Enzyme

The protocol described by Neves et al.^25^ for the biomimetic oxidation of carbamazepine was used as a starting point. After reproducing the experiment for carbamazepine (see Section S1 and Figure S1 in the Supporting Information), the protocol was adapted for the oxidation of CPA and BaP. The reaction mixtures for the two chemicals were prepared individually by mixing an aliquot of a stock solution of the test chemical (0.14 M in acetonitrile for CPA and 0.08 M in dichloromethane for BaP) with stock solutions containing the individual reagents TDCPP (1.02 mM in acetonitrile), ammonium acetate (1.30 M in water), and hydrogen peroxide (30% aqueous solution, equals 9.79 M) in a consecutive manner in amber glass 1.5 mL vials (Labsolute) closed with screw caps with silicone/PTFE septa. Acetonitrile was added to achieve a final volume of 100 μL per reaction mixture. The final concentration of the test chemicals in the reaction mixture was 72 mM for CPA and 0.79 mM for BaP. Both reaction mixtures contained the same concentration of the other reagents: 0.12 mM TDCPP, 143 mM ammonium acetate, and 143 mM hydrogen peroxide. Control samples containing only the test chemical but no reagents were prepared for instrumental analysis. In addition, blank reaction mixtures containing all reagents (including hydrogen peroxide) but no test chemicals were prepared for the bioassay. All reaction mixtures were incubated for 15 min at 30 °C and 1000 rpm on a Bioshake iQ orbital shaker from QInstruments (Jena, Germany). For chemical analysis, the reaction mixtures were injected directly without further dilution or sample preparation for the quantification of the formed metabolites and diluted 1:1000 for the quantification of CPA and BaP.

### Cell Culture and *In Vitro* Bioassay Protocol

The CellSensor p53RE-*bla* HCT-116 cells were obtained from Thermo Fisher (cat. no. K1640) and cultured in T75 flasks with a Matrigel (Corning) coating. The culture medium was McCoýs 5A Medium (Gibco) supplemented with 10% dialyzed FBS (d-FBS, Gibco), 100 U/mL penicillin–streptomycin (Gibco), and 5 μg/mL blasticidin (Applichem). Cells were passaged twice a week and subcultured not more than 25 times after thawing.

The workflow of the p53 bioassay is shown in Figure S2. Per well 15,000 cells were seeded to a black 96-well plate with poly-D-lysine coating (Greiner) using a MultiFlow dispenser from Biotek (cell plate). The seeding medium was Opti-MEM (Gibco) supplemented with 2% dialyzed FBS (Gibco), 1 mM sodium pyruvate (Gibco), 0.1 mM nonessential amino acid solution (Gibco), and 100 U/mL penicillin–streptomycin (Gibco). The medium volume was 100 μL per well. After seeding, cells were incubated for 24 h at 37 °C, 5% CO_2_, and 100% relative humidity for cell attachment. Before the chemicals were dosed to the cells, the confluency of the cells was measured using an IncuCyte S3 life-cell analysis system (Essen Bioscience).

Dosing solutions (i.e., spiked exposure medium) of all test chemicals and reaction mixtures were prepared in 4 mL amber glass vials. The exposure medium was Opti-MEM (Gibco) supplemented with 2% charcoal-stripped FBS (cs-FBS, Gibco) and 100 U/mL penicillin–streptomycin (Gibco). On each plate, mitomycin C was dosed at least once as a reference compound. A stock solution of mitomycin C was prepared in methanol at 1.07 mM and diluted in the exposure medium. The highest concentration of mitomycin C dosed to the cells was 1.65 × 10^–6^ M. For testing the effects of CPA and BaP without metabolic activation, an aliquot of a stock solution of the test chemical in acetonitrile (0.14 M for CPA and 0.79 mM for BaP) was added to a 1.5 mL glass vial, the solvent was evaporated using a nitrogen stream, 1 mL of exposure medium was added, and the vial was vortexed thoroughly to resuspend the chemicals. The highest concentration dosed to the cells was 3.4 × 10^–3^ M for CPA and 2.0 × 10^–5^ M for BaP. In addition, the carcinogenic BaP metabolite BPDE was tested individually using the same dosing procedure, as described for BaP. The highest concentration dosed to the cells was 3.77 × 10^–5^ M for BPDE.

The preparation of the dosing solutions with the abiotic CYP enzyme and liver microsomes is described in detail in the following sections. All dosing solutions were diluted serially nine times in a 96-deep well plate (Polypropylene deep well plate, Corning). For the final step of dosing the chemicals to the cells, 50 μL of seeding medium was removed from each well of the cell plate, and subsequently 170 μL from the prepared dosing plate was added, leading to a total medium volume of 220 μL per well.

After incubating the dosed cell plate for 24 h at 37 °C, 5% CO_2_, and 100% relative humidity, the confluency of the cells was measured again using the Incucyte S3. For the detection of p53 activation, the ToxBLAzer working solution was prepared according to the protocol provided by Thermo Fisher. From each well, 200 μL of medium was aspirated and discarded, and 80 μL of fresh exposure medium and 20 μL of the ToxBLAzer working solution were added to each well. The plate was covered with a black lid to protect it from light and evaporation and centrifuged briefly at 100*g* for max 1 min. Fluorescence was measured immediately (t0h) and after 2 h of incubation at room temperature (t2h) at three different excitation/emission wavelengths (blue: 409/460 nm, green: 409/530 nm, and red: 590/665 nm).

### Preparation of Dosing Solutions Containing Abiotic CYP Enzyme for Metabolic Activation and Hydrogen Peroxide Removal

Reaction mixtures for CPA and BaP were prepared as described in the section “Protocol for oxidation of test chemicals by abiotic CYP enzyme”. An aliquot of the reaction mixtures (24.9–43.1 μL for CPA and 8.2–32.7 μL for BaP) was transferred to a 1.5 mL glass vial, and the solvent was evaporated using a nitrogen stream, 1 mL of exposure medium was added, and the vial was vortexed thoroughly to resuspend the chemicals. For hydrogen peroxide removal, 10 μL of catalase solution in PBS (1239 U/mL) was added to each dosing vial, and the vials were incubated for 15 min at 30 °C and 1000 rpm on a Bioshake iQ orbital shaker from QInstruments (Jena, Germany) before preparing the dosing plates.

### Efficiency of Hydrogen Peroxide Removal

Hydrogen peroxide from the reaction mixtures was found to increase the cytotoxicity and p53 activation of the reaction mixtures, potentially interfering with the detection of the p53 activation of the test chemicals and their respective metabolites. Therefore, catalase was used to degrade the hydrogen peroxide before the reaction mixtures were dosed to the cells. The efficiency of the catalase treatment was assessed by quantifying the hydrogen peroxide concentration of a dosing solution containing 21.5 μL of a blank reaction mixture and 500 μL of exposure medium before and after catalase addition using a Fluorimetric Hydrogen Peroxide Assay Kit from Sigma-Aldrich (MAK165). The assay was conducted according to the instructions given in the product information sheet.

### Preparation of Dosing Solutions Containing Liver Microsomes for Metabolic Activation

Liver microsome solutions (20 mg/mL) were slowly thawed on ice. An aliquot of the liver microsomes was heat-inactivated by incubating the microsomes at 56 °C for 3 h and used as a negative control. Dosing solutions were prepared by adding an aliquot of a stock solution of the test chemical in acetonitrile or methanol (0.14 M for CPA and 0.79 mM for BaP) to a 1.5 mL glass vial, the solvent was evaporated using a nitrogen stream, 1 mL of exposure medium was added, and the vial was vortexed thoroughly to resuspend the chemicals. The highest concentration dosed to the cells was 3.3 × 10^–3^ M for CPA and 2.0 × 10^–4^ M for BaP. NADPH was added at a final concentration of 0.25 g/L, and the vials were vortexed again. Liver microsomes were added last at a final concentration of 0.25 g/L. Blank microsome dosing solutions containing only active liver microsomes and NADPH, but no test chemicals, were prepared. After the microsomes were added, all vials were gently turned over by hand and incubated for 45 min at 37 °C and 450 rpm. The vials were centrifuged at 200*g* for 3 min, and only the supernatant was used for the preparation of the dosing plates.

### Instrumental Analysis

For instrumental analysis, microsome suspensions were extracted using two different techniques. CPA is a hydrophilic drug with a logarithmic octanol–water partition constant (log *K*_ow_) of only 0.63^26^ and therefore mostly freely dissolved in the water phase of the microsome suspensions. Three samples containing liver microsomes, NADPH (concentration 0.25 g/L of each), and CPA (2.2 × 10^–3^ M) in 500 μL of PBS were incubated 45 min at 37 °C (450 rpm, Bioshake) and centrifuged at 200*g* for 3 min. Three control samples were prepared, containing only CPA and PBS and treated analogously. PBS was used instead of the exposure medium to avoid matrix effects. From each sample, 50 μL of the supernatant was transferred to a new vial and mixed for 2 min at 1000 rpm with 250 μL of cold precipitation buffer (90/10 acetonitrile/water with 0.1% formic acid) and incubated for 30 min on ice to precipitate any remaining proteins in the solution. The samples were centrifuged again for 200*g* for 3 min, and 100 μL of the supernatant was transferred to a new vial. The solution was measured directly for the detection of metabolites and diluted 1:10 for the quantification of CPA.

BaP is very hydrophobic (log *K*_ow_ 6.13)^27^ and highly bound to proteins and lipids in the microsome suspension. Therefore, BaP and its metabolites were extracted from the whole microsome suspension. Three samples were prepared in 1.5 mL of PBS with liver microsomes, NADPH (concentration 0.25 g/L of each), and BaP (1.0 × 10^–4^ M) and incubated for 45 min at 37 °C (250 rpm, orbital shaker). Control samples contained 0.25 g/L of heat-inactivated liver microsomes. 1.5 mL of ethyl acetate was added to each sample and shaken horizontally for 15 min (250 rpm, orbital shaker). To improve phase separation, samples were centrifuged at 200*g* for 3 min, and 600 μL of the supernatant was transferred to a new vial. Ethyl acetate was evaporated completely by a nitrogen stream, and the analytes were reconstituted in 60 μL acetonitrile. The solution was measured directly for the detection of metabolites and diluted 1:200 for the quantification of BaP.

Instrumental analysis of carbamazepine is described in Section S2. The chemical concentration of CPA was measured in the reaction mixtures and the extracted liver microsome samples using a liquid chromatography instrument (LC, Agilent 1260 Infinity II) equipped with a Kinetex 1.7 μm, C18, 100 Å, LC column (50 × 2.1 mm) from Phenomenex operating at 25 °C coupled to a triple quadrupole mass spectrometer (MS, Agilent 6420 Triple Quad). Gradient elution at 0.5 mL/min was applied. The eluent was a mixture of acetonitrile and water with 0.1% formic acid, and gradient elution was applied. CPA and its metabolites were ionized using ESI in positive mode with a gas temperature of 320 °C, a gas flow at 8 L/min, a nebulizer at 35 psi, and a capillary voltage of 5000 V. Fragmentor voltage was set to 152 V. The first 3.9 min of each run, the MS was measuring in SIM mode at a *m*/*z* of 277 to detect 4-hydroxycyclophosphamide (4OH-CPA)/aldophosphamide. An MRM method was used for the quantification of CPA from 3.9 to 6 min, and *m*/*z* of 261 was used as a precursor ion. The quantifier ion had *m*/*z* 140 and the qualifier ion *m*/*z* 63.1; collision energies were 22 and 38 V, respectively.

BaP and its metabolites were separated on a Kinetex 3.5 μm, PAH LC column (50 × 2.1 mm) from Phenomenex at 25 °C using the same LC instrument but coupled to a diode array (330 nm detection wavelength) and a fluorescence detector (excitation at 260 nm, emission wavelength at 420 nm). Gradient elution at 0.8 mL/min was applied. The eluent was a mixture of acetonitrile and water. Standard solutions of CPA (5–50,000 ng/mL) and BaP and its metabolites (1–2000 ng/mL) in acetonitrile were measured together with the samples and used for quantification.

### Data Evaluation

A linear model was used to derive the effect concentrations for cytotoxicity and activation of p53.^28^ The cell viability from the confluency measurements was calculated by dividing the confluency of the samples by the average confluency of the negative controls on the plate. The cell viability was also calculated from the red fluorescence signal at 665 nm of the ToxBLAzer reagent (*F*_665nm_) using eq 1
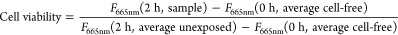
1

The concentration, which led to a reduction of the cell viability of 10% (IC_10_) was calculated from the slope of the linear range of the concentration–response curves (CRCs, eq 2) for both measurements, confluency and ToxBLAzer reagent^28^
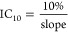
2

The reference compound in the p53 assay was mitomycin C. The induction ratio (IR) was calculated by comparing the blue/green (B/G) ratio of the samples to the signal of the unexposed cells. The concentration, which led to an IR of 1.5 (EC_IR1.5_) was used as an activity benchmark and was calculated from the linear range of the CRCs using eq 3
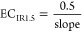
3

The specificity ratio (SR) for the activation of p53 was calculated by eq 4 using the IC_10_ derived from the ToxBLAzer measurement.^29^ The ToxBLAzer measurements were used for the calculation because cytotoxicity was often not detected by the confluency measurements
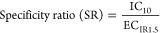
4

## Results

### Metabolism of CPA and BaP by Abiotic CYP Enzyme and Liver Microsomes

**Figure 2:**
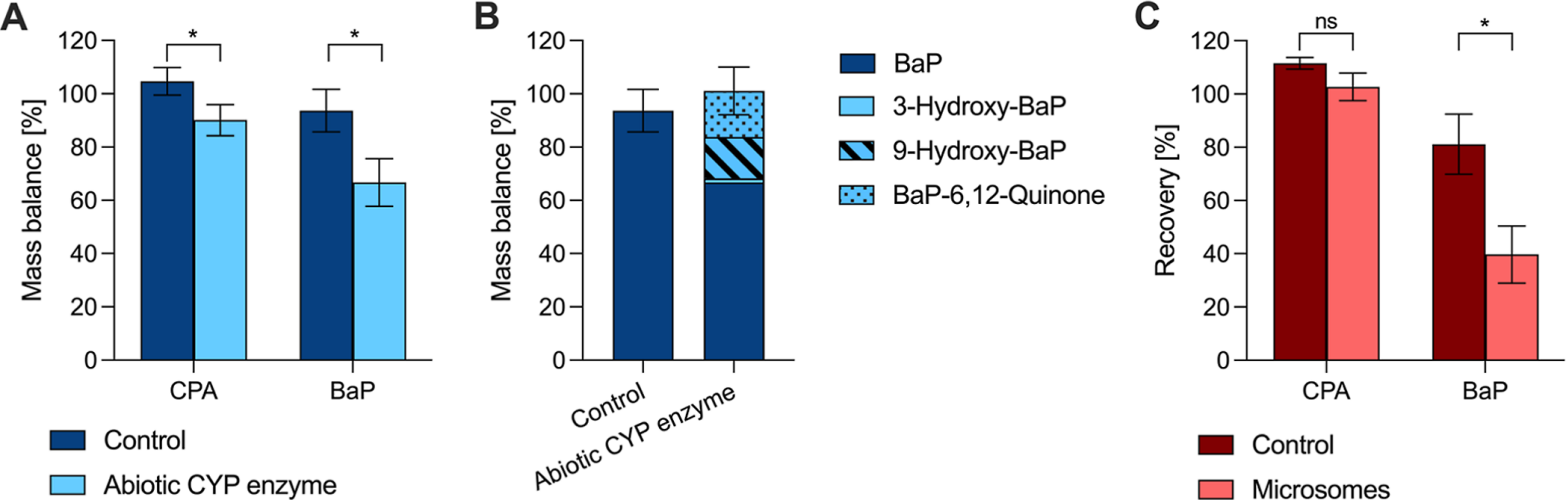
(A) Mass balance of reaction mixtures with abiotic CYP enzyme for cyclophosphamide (CPA) and benzo[*a*]pyrene (BaP), (B) mass balance of reaction mixtures with abiotic CYP enzyme for BaP, including the detected metabolites, and (C) recovery of CPA and BaP from microsome suspensions.

The concentration of CPA and BaP was reduced significantly due to oxidation by the abiotic CYP enzyme (unpaired *t*-test, p value 0.03 for BaP and 0.02 for CPA, Figure 2A). As expected, the mass balance of the control samples was excellent, 104.7 ± 5.2% for CPA and 93.7 ± 8.0% for BaP (Figure 2A) because no sample preparation other than a 1000-fold dilution was necessary before instrumental analysis.

In the reaction mixtures of CPA, an additional peak at *m*/*z* 277 was found that was not present in the control samples. It was not possible to confirm the identity of the metabolite or quantify its amount because no analytical standard was available for 4OH-CPA or aldophosphamide. However, the peak area of the suspected metabolite was similar in all the tested reaction mixtures (*n* = 6 in total). The ratio between the peak area of the metabolite and the peak area of CPA in the reaction mixtures was 0.05 ± 0.01. This low ratio of peak areas, together with the fact that 90.1 ± 5.9% of CPA added was still found after the reaction with the abiotic CYP enzyme, indicates that only a small fraction of CPA was metabolized. For BaP, 66.7 ± 8.9% of the nominal amount added was found in the reaction mixtures upon oxidation by the abiotic CYP enzyme. Several metabolites were detected in the reaction mixtures (Figures S3 and S4). Based on their retention times, three peaks were allocated as 3-hydroxybenzo[*a*]pyrene (M1), 9-hydroxybenzo[*a*]pyrene (M2), and benzo[*a*]pyrene-6,12-quinone (M7), respectively. The quantified amounts of BaP, M1, M2, and M7 were summed up, and the resulting mass balance was 101.1 ± 9.0% (Figure 2B), indicating that the major oxidation products were identified.

For CPA, no metabolism by the liver microsomes could be detected by instrumental analysis because recovery from the PBS controls (111.6 ± 2.2%) and the samples containing active rat liver microsomes and NADPH (102.7 ± 5.2%) was not significantly different (unpaired *t*-test, *p* value 0.08, Figure 2C). Furthermore, no peaks were detected at *m*/*z* = 277. For BaP, the recovery from the control samples containing inactive liver microsomes and NADPH (81.2 ± 11.3%) was significantly higher compared to the recovery from the samples containing the active rat liver microsomes and NADPH (39.7 ± 10.7%), indicating the metabolism of BaP (unpaired *t*-test, *p* value 0.01, Figure 2C). Unfortunately, no further analysis of the formed metabolites was possible for BaP because no additional peaks were detected in the microsome extracts compared with the control samples.

### Cytotoxicity and p53 Activation of Positive Control Samples and Blanks and Efficiency of Hydrogen Peroxide Removal

The positive control mitomycin C showed a stable and highly specific activation of p53 (Figure S5 and Table 1) with an EC_IR1.5_ of 4.96 × 10^–8^ M and a SR of 22. Cytotoxicity was also detected reproducibly at the highest tested concentrations. The IC_10_ for cytotoxicity was 1.11 × 10^–6^ M determined from the ToxBLAzer reagent, and 1.08 × 10^–6^ M determined from the confluency measurements.

**Table 1:** Effect Concentrations for Cytotoxicity (IC_10_) and Activation of p53 (EC_IR1.5_) for All of the Test Chemicals

chemical	cytotoxicity based on confluency		cytotoxicity based on ToxBLAzer		activation of p53		SR
	IC_10_ (M)	CV	IC_10_ (M)	CV	EC_IR1.5_ (M)	CV	
*Mitomycin C*^a^	1.08 × 10–^6^	6.2%	1.11 × 10–^6^	9.7%	4.96 × 10–^8^	2.1%	22.4
*CPA*							
without activation	not cytotoxic up to 3.36 × 10–^3^		2.41 × 10–^3^	24.2%	not active up to 3.36 × 10–^3^		
active microsomes	3.90 × 10–^4^	13.1%	2.17 × 10–^4^	7.2%	4.10 × 10–^5^	6.3%	5.3
inactive microsomes	not cytotoxic up to 3.36 × 10–^3^		2.37 × 10–^3^	28.7%	1.91 × 10–^3^	7.2%	1.2
abiotic CYP enzyme	2.08 × 10–^4^	17.3%	9.80 × 10–^5^	12.5%	2.70 × 10–^5^	7.0%	3.6
*BaP*							
without activation	not cytotoxic up to 2.00 × 10–^5^		1.37 × 10–^5^	39.3%	not active up to 2.00 × 10–^5^		
active microsomes	not cytotoxic up to 2.00 × 10–^4^		1.85 × 10–^4^	70.1%	7.33 × 10–^5^	5.4%	2.5
inactive microsomes	not cytotoxic up to 2.00 × 10–^4^		not cytotoxic up to 2.00 × 10^–4^		4.13 × 10^–5^	11.1%	
abiotic CYP enzyme	6.90 × 10–^6^	45.6%	1.71 × 10–^6^	15.4%	not active up to 5.00 × 10^–6^		
*BPDE*	3.70 × 10–^6^	9.9%	4.74 × 10–^6^	17.9%	5.96 × 10^–6^	7.0%	0.8

a Fitted from all experiments conducted for the present study (19 replicates in total). CPA - cyclophosphamide. BaP - benzo[a]pyrene. BPDE - benzo[*a*]pyrene-7,8-dihydrodiol 9,10-epoxide. CV - coefficient of variance. SR - specificity ratio (eq 4).

The blank microsome samples containing active liver microsomes, NADPH, but no test chemicals did not show any cytotoxic effects and no activation of p53 (Figure S6A). However, the IR for p53 activation increased at the highest dose concentrations, nearly exceeding the activity threshold of 1.5.

Before the catalase was added, the dosing solution of the reaction mixtures prepared in exposure medium was found to contain 5.3 mM hydrogen peroxide, which was slightly less than the nominal amount added (6.2 mM). This means that only a small fraction of hydrogen peroxide was degraded during the reaction with the abiotic CYP enzyme. After catalase treatment, the concentration decreased to 0.002 mM, which means that the dosing solutions applied to the cells contained only trace amounts of hydrogen peroxide that should not have caused any adverse effects. This is supported by the fact that the blank reaction mixtures containing all reagents for the abiotic CYP enzyme but no test chemical and that were treated with catalase for hydrogen peroxide removal showed no cytotoxicity and no activation of p53 (Figure S6B).

### Cytotoxicity and p53 Activation of CPA and BaP with and without Metabolic Activation

**Figure 3:**
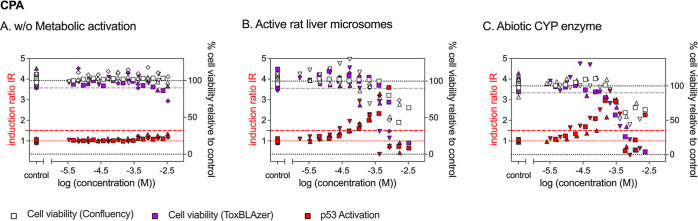
Concentration–response curves from the p53 bioassays for cyclophosphamide (CPA) (A) without metabolic activation, (B) activated with rat liver microsomes, and (C) activated with abiotic CYP enzyme. Different symbols indicate different experimental replicates.

**Figure 4:**
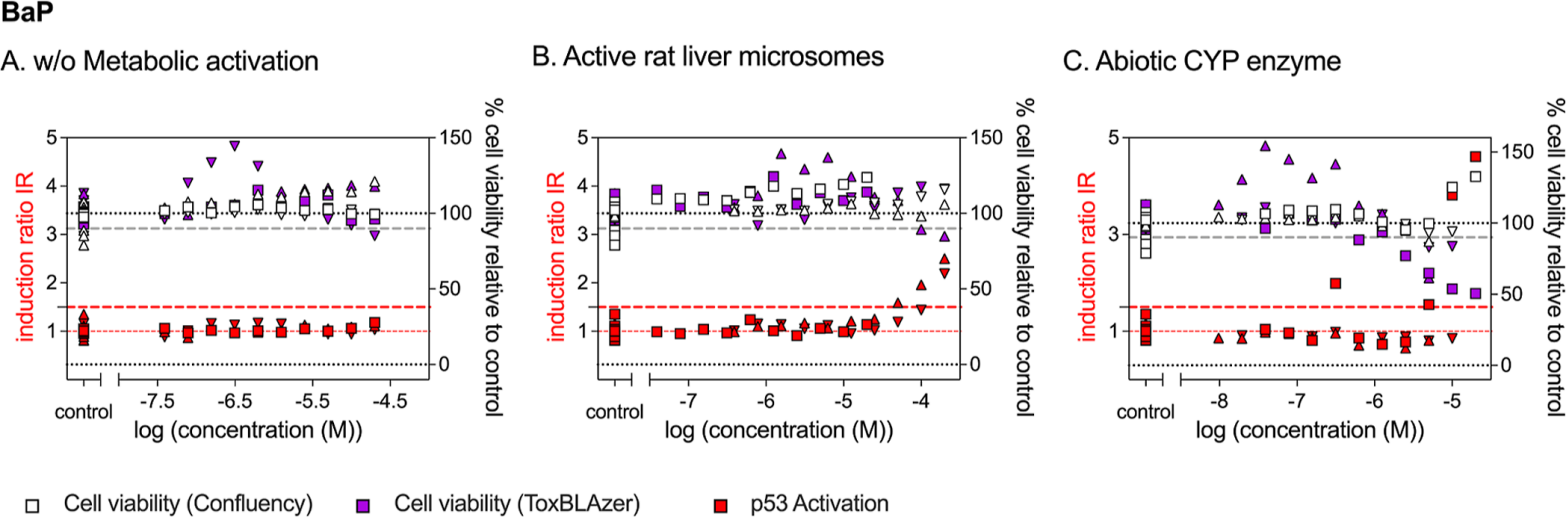
Concentration–response curves from the p53 bioassays for benzo[a]pyren (BaP) (A) without metabolic activation, (B) activated with rat liver microsomes, and (C) activated with abiotic CYP enzyme. Different symbols indicate different experimental replicates.

All concentration response curves of CPA are shown in Figure S7, a summary is presented in Figure 3, and all concentration response curves of BaP and BPDE are shown in Figure S8 and summarized in Figure 4. The derived effect concentrations for cytotoxicity (IC_10_) and activation of p53 (EC_IR1.5_) for all test chemicals can be found in Table 1.

CPA showed no activation of p53 without metabolic activation (Figure 3A). Cytotoxicity was detected with the ToxBLAzer reagent at the highest dosed concentrations (IC_10_ 2.41 × 10^–3^ M), but not with the confluency measurements. Incubation of CPA with active rat liver microsomes increased the cytotoxicity of CPA by a factor of 11 (IC_10_ 2.17 × 10^–4^ M) and led to a strong activation of p53 with an EC_IR1.5_ of 4.10 × 10^–5^ M (Figure 3B). Incubation with inactive liver microsomes (see also Figure S7C) did not increase cytotoxicity of CPA (IC_10_ 2.37 × 10^–4^ M) and only caused an activation of p53 close to cytotoxic concentrations (EC_IR1.5_ of 1.91 × 10^–3^ M). The reaction with the abiotic CYP enzyme also increased the cytotoxicity of CPA by a factor of 25 (IC_10_ 9.80 × 10^–5^ M) and caused a strong activation of p53 with an EC_IR1.5_ of 2.70 × 10^–5^ M (Figure 3C). The effect concentrations obtained with both metabolization systems were very close, within a factor of 2 for cytotoxicity and 1.5 for p53 activation.

No activation of p53 was detected for BaP without metabolic activation (Figure 4A). Cytotoxicity was detected with the ToxBlazer reagent (IC_10_ 1.37 × 10^–5^ M), but was not measurable with the confluency measurements. Interestingly, the response of the ToxBlazer reagent showed an increase at low concentrations, which may indicate an increase in cellular esterase activity or metabolism in general. Incubation of BaP with active rat liver microsomes did not cause any cytotoxicity if the same dosing concentration was used as in the experiments without microsomes (square symbols in Figure 4B). Using the *in vitro* exposure model from Fischer et al.,^30^ the freely dissolved (i.e., bioavailable) fraction (*f*_free_) of BaP in the exposure medium was calculated with and without microsomes. Because BaP shows strong partitioning to membrane lipids, the *f*_free_ was calculated to be 0.39% without and only 0.03% with 0.25 mg/mL liver microsomes. Consequently, dosing concentrations were increased by a factor of 10. The IC_10_ for cytotoxicity with active liver microsomes was 1.85 × 10^–4^ M, and therefore a factor of 13 higher than without adding the microsomes. Considering the lower *f*_free_ in the samples containing microsomes, cytotoxicity is not decreased by incubation with the microsomes but is comparable. The activation of p53 was detected for BaP upon incubation with active and inactive microsomes at similar concentrations (Figures 4B and S8B,C). The EC_IR1.5_ was 7.33 × 10^–5^ and 4.13 × 10^–5^ M, respectively. This indicates that the activation of p53 was probably not caused by the formed BaP metabolites but was rather an artifact caused by the microsomes or NADPH. The activation of BaP with the abiotic CYP enzyme caused an increase in cytotoxicity by a factor of 8, but no activation of p53 was detected. The genotoxic metabolite BPDE showed 29 times higher cytotoxicity than BaP (IC_10_ 4.47 × 10^–6^ M). The activation of p53 was detected for BPDE, but only close to cytotoxic concentrations (ToxBLAzer, Figure S8E) and therefore with low specificity (SR 0.8).

## Discussion

### Metabolism and Genotoxicity of CPA

**Figure 5:**
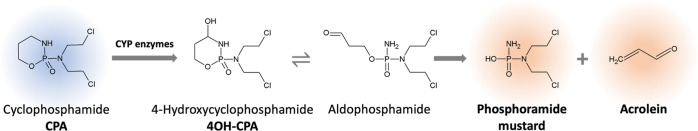
Metabolic activation of cyclophosphamide (CPA) in vivo. (Geno)toxic metabolites are indicated by an orange glow.

CPA is a hydrophilic prodrug used in chemotherapy. In vivo, CPA is activated in the liver by CYP enzymes (mainly CYP2B, but also other CYP isoenzymes, see also Figure 5)^31^ by hydroxylation to 4OH-CPA. 4OH-CPA is in equilibrium with its ring-opened tautomer aldophosphamide, which spontaneously degrades to the actual (geno) toxic metabolites, phosphoramide mustard and acrolein.^32^

In the experiments of the present study, we were not able to confirm the formation of the genotoxic metabolites by instrumental analysis because no analytical standard of 4OH-CPA was available. Phosphoramide mustard and acrolein are even more difficult to detect because of their high reactivity and low molecular weight (acrolein). However, we found that the abiotic CYP enzyme reproducibly led to the formation of a product with a *m*/*z* of 277, which could have been 4OH-CPA or aldophosphamide. No peaks were detected at a *m*/*z* of 277 in the microsome extracts of CPA, and most likely, the unstable 4OH-CPA was lost during sample preparation. In the p53 bioassay, strong activation of p53 and increased cytotoxicity was observed with both metabolization systems. Hence, it can be concluded that the genotoxic metabolites were formed with rat liver microsomes and the abiotic CYP enzyme. Because the effect concentrations obtained with both metabolization systems agreed within a factor of 2 for cytotoxicity and 1.5 for p53 activation, the yields of the genotoxic metabolites seem to be comparable.

### Metabolism and Genotoxicity of BaP

**Figure 6:**
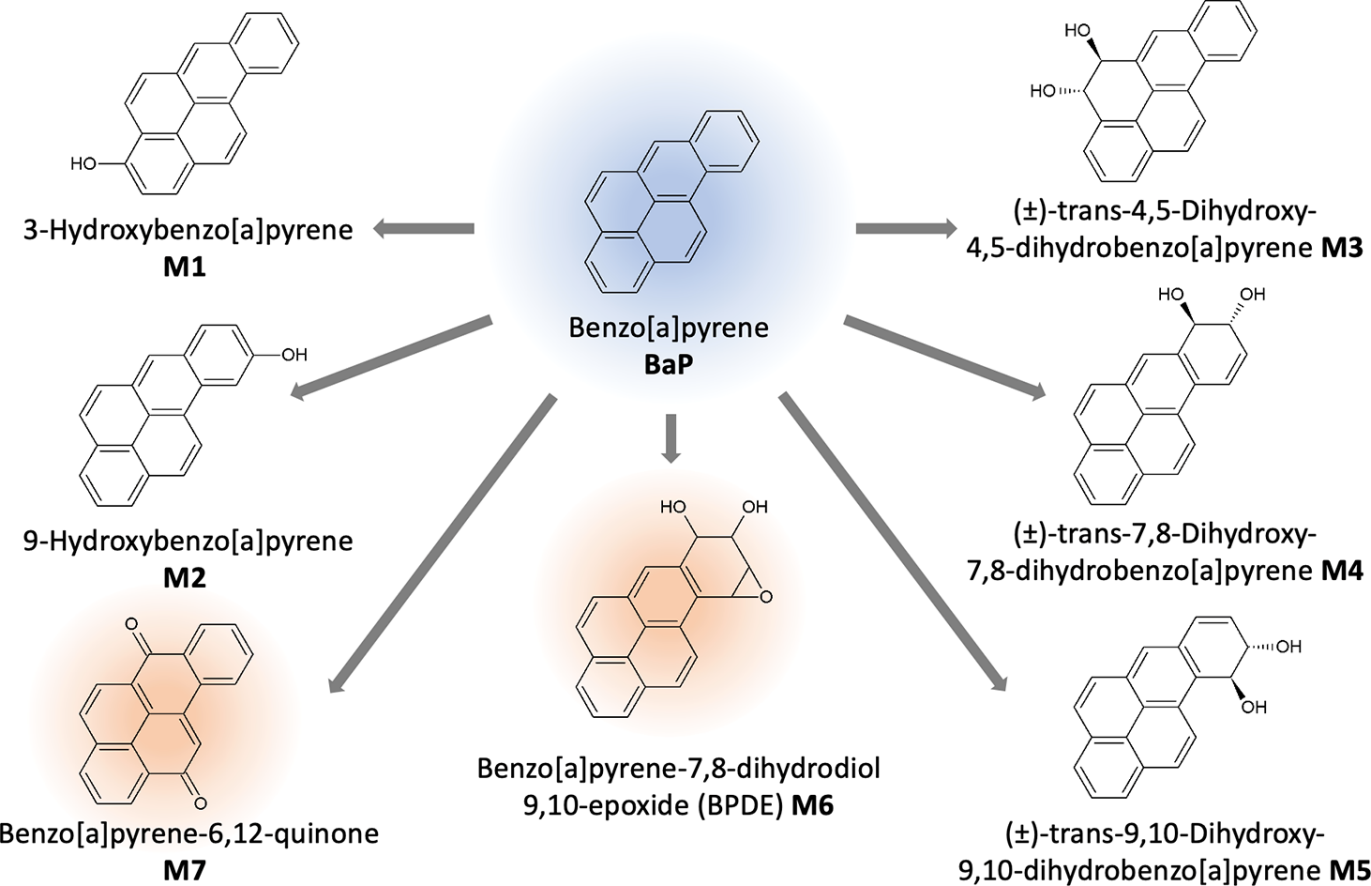
Benzo[a]pyrene (BaP) and its metabolites that were included in the instrumental analysis of the present study. Genotoxic (M6) and cytotoxic (M7) metabolites^33^ are indicated by an orange glow.

BaP is a PAH that is metabolized in vivo to a variety of different metabolites, including epoxides, dihydrodiols, phenols, and quinones.^33^ First, epoxides are formed by P450 monooxygenases (BaP 2,3-, 4,5-, 7,8-, and 9,10-epoxide). Subsequent metabolism by epoxide hydrolase leads to the formation of the corresponding diols (metabolites M3, M4, and M5 in Figure 6). Monohydroxylated metabolites like 3- and 9-OH BaP (M1 and M2 in Figure 6) are mainly formed by the nonenzymatic rearrangement of the epoxides.^33^ Oxidation of the primary metabolites leads to the formation of different secondary metabolites like BPDE (M6 in Figure 6), which is considered highly mutagenic and carcinogenic.^34^ BaP quinones (e.g., BaP 6,12-quinone, M7 in Figure 6) are formed by the autoxidation of 6-OH BaP and can redox cycle between their corresponding hydroquinones and semiquinone radicals, leading to the formation of reactive oxygen species.^33^

Unfortunately, no metabolites could be quantified in the microsome extracts in the present study. Compared to previous analytical studies, the concentration of the microsomes in the present study was much lower (0.25 g/L compared to 1 g/L), which may have led to a low yield of metabolites. Microsome concentrations >0.25 g/L were not tested because they caused significant cytotoxicity in the p53 bioassay. Previous studies using rat liver microsomes for metabolism studies with BaP mainly found 3-hydroxybenzo[*a*]pyrene (M1) and other monohydroxylated BaP metabolites, as well as diols and some quinones (diones).^35^ Because the cytotoxicity of BaP was similar or lower after metabolism with rat liver microsomes (see also Table 1 and Figure 4), it can be assumed that mainly monohydroxylated BaP metabolites and diols were formed that are less toxic compared to BaP.^33^ The main oxidation products of BaP formed by the abiotic CYP enzyme were 9-hydroxybenzo[*a*]pyrene (M2) and benzo[*a*]pyrene-6,12-quinone (M7, Figure 2B). Additionally, a small amount of 3-hydroxybenzo[*a*]pyrene (M1) was detected. More peaks can be seen in the chromatograms of the reaction mixtures (Figures S3 and S4) that could not be identified. Due to their close retention times, it can be assumed that those metabolites are other monohydroxylated BaP metabolites and quinones. The genotoxic metabolite BPDE could not be detected in the reaction mixtures. A previous study that applied a similar biomimetic catalyst for the production of PAH metabolites as used in the present study also identified benzo[*a*]pyrene-6,12-quinone and other quinones as the most abundant oxidation products of BaP.^23^ The formation of the quinones also explains the increase in cytotoxicity observed in the p53 bioassay upon oxidation with the abiotic CYP enzyme. The quinone metabolites of PAHs are known to cause cytotoxicity by various mechanisms, including the formation of reactive oxygen species and DNA damage.^36^ The activation of p53 was not observed in the present study but could have been masked by cytotoxicity in the experiments with the abiotic CYP enzyme (see also Figure 4C). The metabolization with liver microsomes also did not lead to a specific activation of p53 in the present study. For both metabolization systems, it needs to be considered that metabolism takes place in the assay medium, and reactive metabolites like BPDE may form adducts with proteins like serum albumin^37^ before they can enter the cells and cause adverse effects.

Even BPDE, which is considered the ultimate carcinogenic metabolite of BaP,^33^ did not show a highly specific activation of p53, indicating that the used assay system is not ideal to detect the genotoxic potential of BaP and its metabolites. Other genotoxicity assays may be more appropriate to further explore the use of the abiotic CYP enzyme as a replacement for rat liver microsomes or S9 fraction, such as the *in vitro* mammalian cell micronucleus assay^8^ or the Ames fluctuation test.^38^

### Advantages and Limitations of Abiotic CYP Enzyme

The abiotic CYP enzyme tested in the present study was successfully applied for the metabolic activation of the two genotoxic reference compounds CPA and BaP. The material is free of any animal-derived components and has a completely chemically defined composition, which makes this approach superior to conventional animal-derived materials such as S9 fractions or microsomes from induced rat livers or humans. The used reagents are also low-cost compared to liver microsomes, and no special equipment is required apart from a high-speed shaker and a nitrogen evaporator. Furthermore, the reagents (acetonitrile, ammonium acetate, TDCPP, and hydrogen peroxide) can either be evaporated (acetonitrile and ammonium acetate) or degraded by catalase (hydrogen peroxide) before the solutions are applied to the cells. Only the catalyst TDCPP itself, remains in the dosing solutions, but it is dosed at several hundred times lower concentrations compared to the test chemicals and showed no cytotoxic effects or activation of p53 in the tested cell line (Figure S6B).

Conveniently, the reaction mixtures can be directly injected into a HPLC system without any sample preparation. Because the solutions can be measured immediately, the detection of reactive or unstable metabolites is also possible. For the present study, only target analysis of the formed metabolites was performed using either a fluorescence detector or a triple-quadrupole MS. Further analysis and identification of the metabolites would require the use of high-resolution MS and suspect or nontarget screening approaches.

The use of induced rat liver microsomes or S9 fraction for the metabolic activation of test chemicals is often problematic because these materials show high lot-to-lot variability and the inducers that are administered to the rats often remain in the final product, potentially causing cytotoxic effects.^9^ The abiotic CYP enzyme avoids both potential artifacts.

Besides all the many advantages, several shortcomings of biomimetic catalysts have been identified in previous studies.^17^ For example, the stereoselectivity of the biomimetic catalysts may be different compared to natural enzymes, which is problematic for the simulation steroid metabolism.^17^ The amount and nature of oxidation products formed are also highly dependent on the type of catalyst and oxygen donor used.^17^ It is therefore unlikely that one combination of a catalyst and oxygen donor will be able to mimic all CYP isoenzyme functions. This limitation may be further explored using established LCMS substrates for different CYP enzymes and their respective metabolites as model compounds. Including abiotic CYP enzymes as metabolization systems in existing test guidelines, such as OECD Test Guideline no. 487, would also require testing of more promutagenic chemicals and genotoxic end points like the formation of micronuclei.

## Supporting Information Available

Protocol for oxidation and instrumental analysis of carbamazepine, workflow of the p53 bioassay, chromatograms of reaction mixtures, and concentration–response curves of mitomycin C, blanks, CPA, and BaP (PDF)
