# Supplementary material for: In Vitro Metabolism and p53 Activation of Genotoxic Chemicals: Abiotic CYP Enzyme vs Liver Microsomes
Source: Chem Res Toxicol. 2024 Jun 20;37(8):1364–73. doi: 10.1021/acs.chemrestox.4c00101 (PMC11337206; doi:10.1021/acs.chemrestox.4c00101)
Supplement: Supplementary file 1 — tx4c00101_si_001.pdf [file tx4c00101_si_001.pdf]

Supporting Information

In vitro Metabolism and p53 Activation of Genotoxic Chemicals: Abiotic CYP Enzyme vs. Liver Microsomes

Luise Henneberger[†,*], Julia Huchthausen[†], Jenny Braasch[†], Maria König[†], Beate I. Escher[†, §]

[†] Helmholtz Centre for Environmental Research – UFZ, Department of Cell Toxicology, Permoserstr. 15, 04318 Leipzig, Germany

[§] Eberhard Karls University Tübingen, Environmental Toxicology, Department of Geosciences, 72076 Tübingen, Germany

Table of Contents

S1. Oxidation and instrumental analysis of carbamazepine 2

S2. Workflow of the p53 bioassay 3

S3. Chromatograms of reaction mixtures 3

S4. Concentration-response curves of mitomycin C 4

S5. Concentration-response curves of blanks 4

S6. Concentration-response curves of cyclophosphamide 5

S7. Concentration-response curves of benzo[a]pyrene 6

References 7

S1. Oxidation of carbamazepine

Following the protocol from Neves et al.[1] 11.81 mg of carbamazepine and 7.5 mg of ammonium acetate were weighed onto an amber glass vial and 90 µL of TDCPP stock solution (1 g/L in acetonitrile) and 900 µL acetonitrile were added. An aliquot of 1 µL was taken from the mixture and diluted in 1 mL acetonitrile for instrumental analysis. To start the reaction 12.5 µL of hydrogen peroxide stock solution (1.76 M in acetonitrile) were added and the vial was incubated at 30°C and 1000 rpm. Aliquots of 1 µL were taken after 15, 30, 45, 60, 75, 90, 105 and 120 min and diluted in 1 mL acetonitrile. After the aliquots were taken 12.5 µL of hydrogen peroxide stock solution (1.76 M in acetonitrile) were added. All diluted aliquots were measured by LC-UV and the responses of carbamazepine and its epoxide were recorded at 220 nm. The response of carbamazepine decreased after every addition of hydrogen peroxide, while the signal of carbamazepine 10,11-epoxide increased (Figure S1 A). No additional peaks were detected, indicating no significant formation of other reaction products.
To simplify the preparation of the reaction mixtures, we tested whether (I) adding an excess of hydrogen peroxide once or twice (each 10 times the concentration of carbamazepine) instead of a stepwise addition would also be sufficient and (II) dilution of the reagents was possible to minimize the final concentration of the reagents in the bioassays medium. Three mixtures were prepared. The first mixture was prepared as described above, using 10 mg carbamazepine, 6.5 mg ammonium acetate, 74 µL of TDCPP stock solution (1 g/L in acetonitrile), 43 µL hydrogen peroxide (30% aqueous solution) and 883 µL acetonitrile. The second reaction mixture contained 1 mg carbamazepine, 0.65 mg ammonium acetate, 7.4 µL of TDCPP stock solution (1 g/L in acetonitrile), 4.3 µL hydrogen peroxide (30% aqueous solution) and 988 µL acetonitrile and the third mixture contained 100 µL carbamazepine stock solution (1 g/L in acetonitrile), 65 µL ammonium acetate stock solution (1 g/L in water), 0.74 µL TDCPP stock solution (1 g/L in acetonitrile), and 2.2 µL hydrogen peroxide stock solution (1.76 M in acetonitrile) and 832 µL ACN. Aliquots of 1 µL from the reaction mixtures were taken before adding the hydrogen peroxide and after 15 min of incubation at 30°C and 1000 rpm and diluted in 1 mL acetonitrile. Hydrogen peroxide was added to all mixtures again and aliquots were taken and diluted after 60 min incubation time in total. The concentrations of carbamazepine and carbamazepine 10,11-epoxide in all diluted aliquots were measured by LC-MS/MS. After the first addition of hydrogen peroxide carbamazepine 10,11-epoxide was only detected in the reaction mixture with 10 g/L carbamazepine and after the second addition in the 10 and 1 g/L mixture (Figure S1 B). For the 10 g/L mixture the concentration of carbamazepine 10,11-epoxide increased compared to the first measurement. No carbamazepine 10,11-epoxide was found in the 0.1 g/L mixture. We concluded from the results that an excess of hydrogen peroxide added only once is sufficient, but dilution of the reagents is not advisable, because reaction kinetics are apparently slowed down significantly.

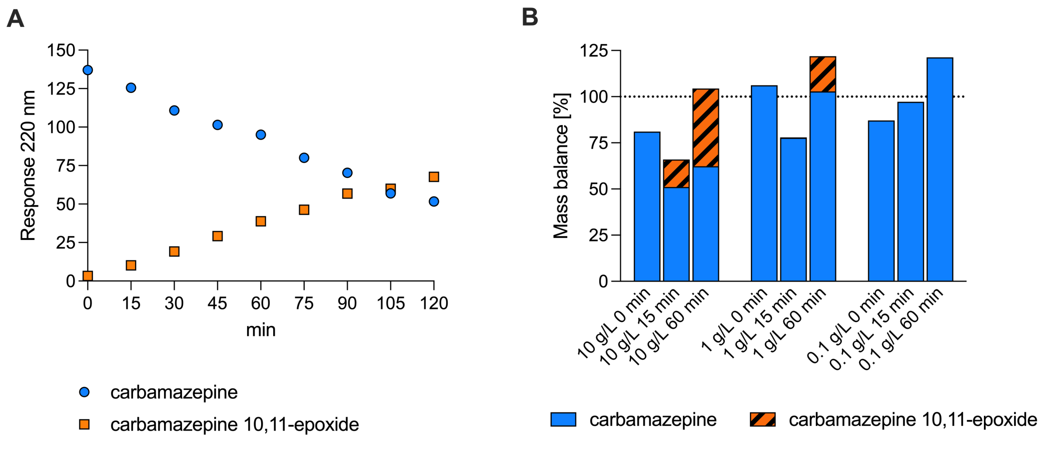

Figure S1. (A) Responses of carbamazepine and carbamazepine 10,11-epoxide over time, (B) Concentration and time dependence of reaction.

S2. Instrumental analysis of carbamazepine

Carbamazepine and carbamazepine 10,11-epoxide were measured using a liquid chromatography instrument (LC, Agilent 1260 Infinity II) equipped with a Kinetex 1.7 µm, C18, 100 Å, LC column (50 × 2.1 mm) from Phenomenex operating at 40°C coupled to a diode array detector (220 nm detection wavelength) and a triple quadrupole mass spectrometer (MS, Agilent 6420 Triple Quad). Gradient elution at 0.5 ml/min was applied. The eluent was a mixture of acetonitrile and water with 0.1% formic acid. Carbamazepine and carbamazepine 10,11-epoxide were ionized using ESI in positive mode with a gas temperature of 300°C, gas flow at 10 L/min, nebulizer at 50 psi and a capillary voltage of 3500 V. An MRM method was used for the quantification of both chemicals. For carbamazepine m/z of 237.1 was used as precursor ion. Fragmentor voltage was set to 130 V. The quantifier ion had m/z 194.1 and the qualifier ion m/z 179.1, collision energies were 16 and 40 V, respectively. For carbamazepine 10,11-epoxide m/z of 253.28 was used as precursor ion. Fragmentor voltage was set to 86 V. The quantifier ion had m/z 236 and the qualifier ion m/z 180.1, collision energies were 6 and 30 V, respectively.

S3. Workflow of the p53 bioassay

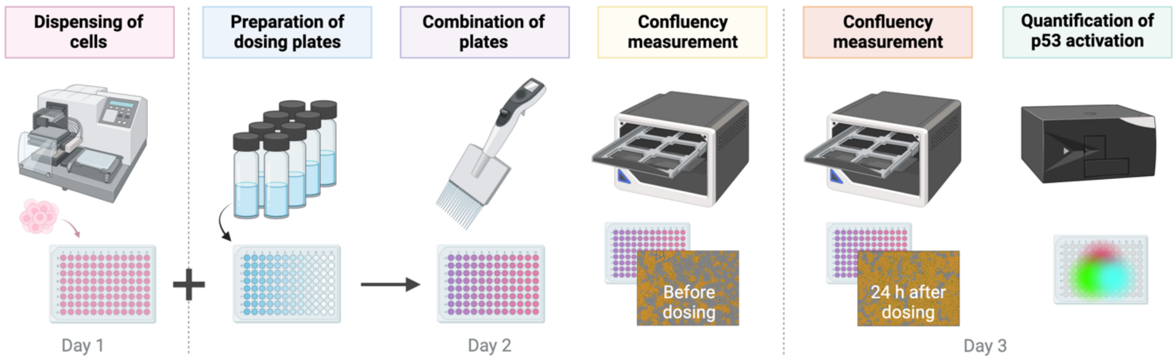

Figure S2. Workflow of the p53 bioassay. Created with BioRender.com.

S4. LC Chromatograms of reaction mixtures

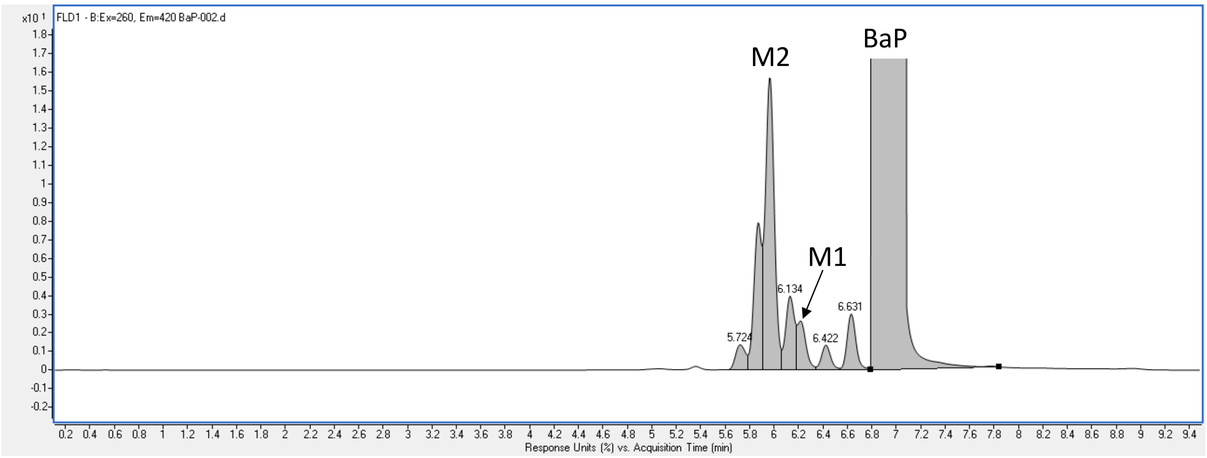

Figure S3. LC Chromatogram of BaP and its metabolites formed with abiotic CYP enzyme, FLD signal 260/420 nm. M1 - 3-hydroxybenzo[a]pyrene, M2 - 9-hydroxybenzo[a]pyrene.

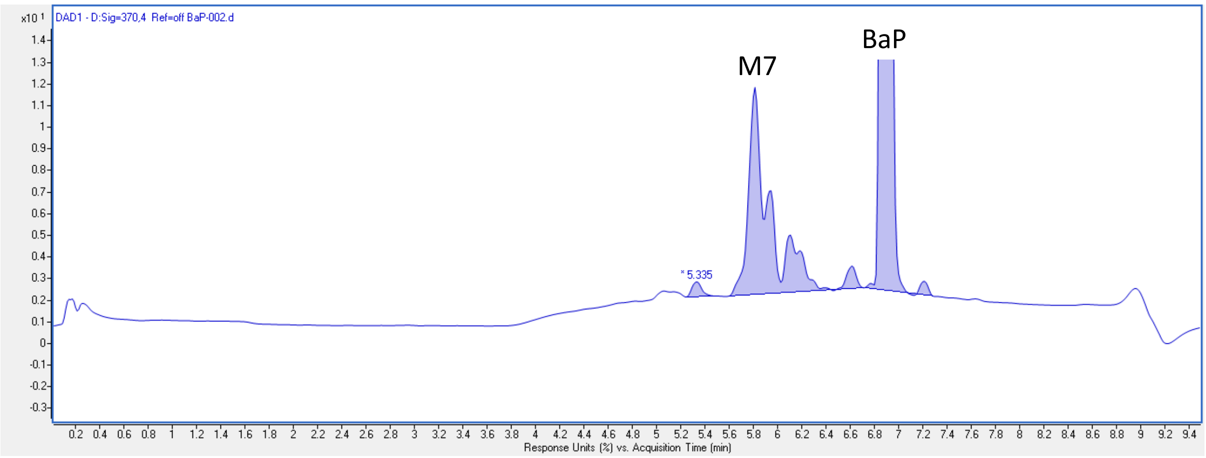

Figure S4. Chromatogram of BaP and its metabolites formed with abiotic CYP enzyme, DAD signal 370 nm. M7 - benzo[a]pyrene-6,12-quinone.

S5. Concentration-response curves of mitomycin C

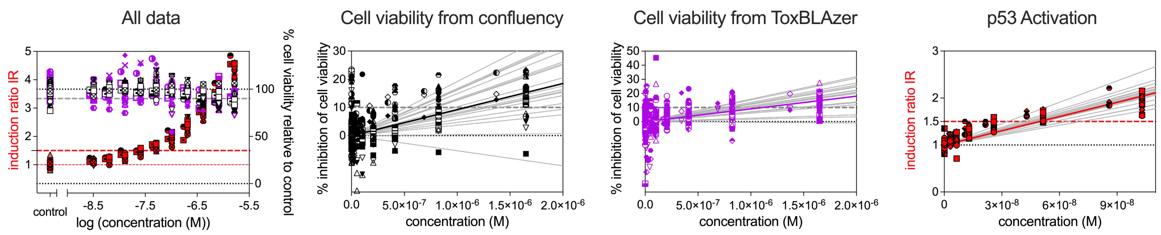

Figure S5. Concentration-response curves of the reference compound mitomycin C. Different symbols indicate different experimental replicates (19 in total).

S6. Concentration-response curves of blanks

A. Active rat liver microsomes + NADPH without test chemicals

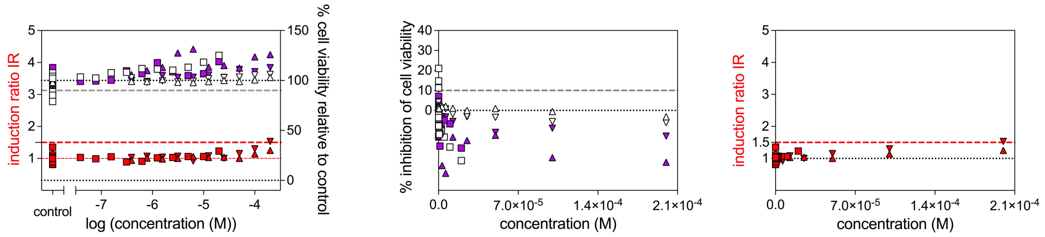

B. Abiotic CYP enzyme without test chemicals

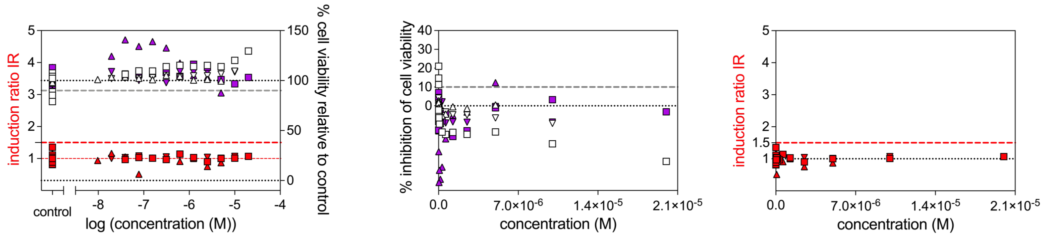

Figure S6. Concentration-response curves of (A) microsome blanks and (B) blank reaction mixtures (+evaporation and catalase treatment). Different symbols indicate different experimental replicates.

S7. Concentration-response curves of cyclophosphamide

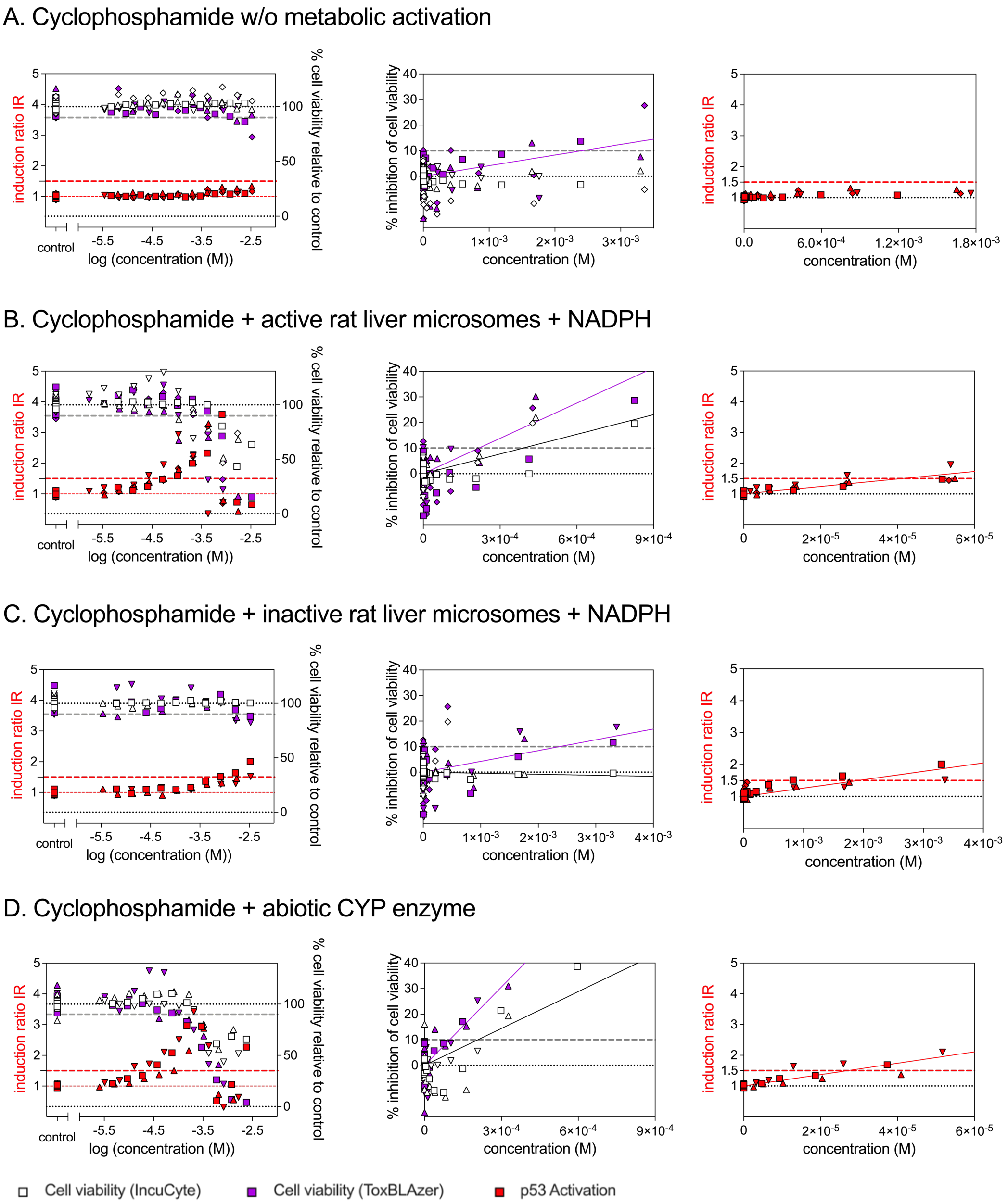

Figure S7. Concentration-response curves of cyclophosphamide (A) without metabolic activation, (B) incubated with active rat liver microsomes and NADPH, (C) incubated with inactive rat liver microsomes and NADPH, (D) activated with abiotic CYP enzyme. Different symbols indicate independent experimental replicates. Left plots: overview of all data on logarithmic scale, middle plots: linear fits of cytotoxicity, right plots: linear fit of p53 activation.

S8. Concentration-response curves of benzo[a]pyrene

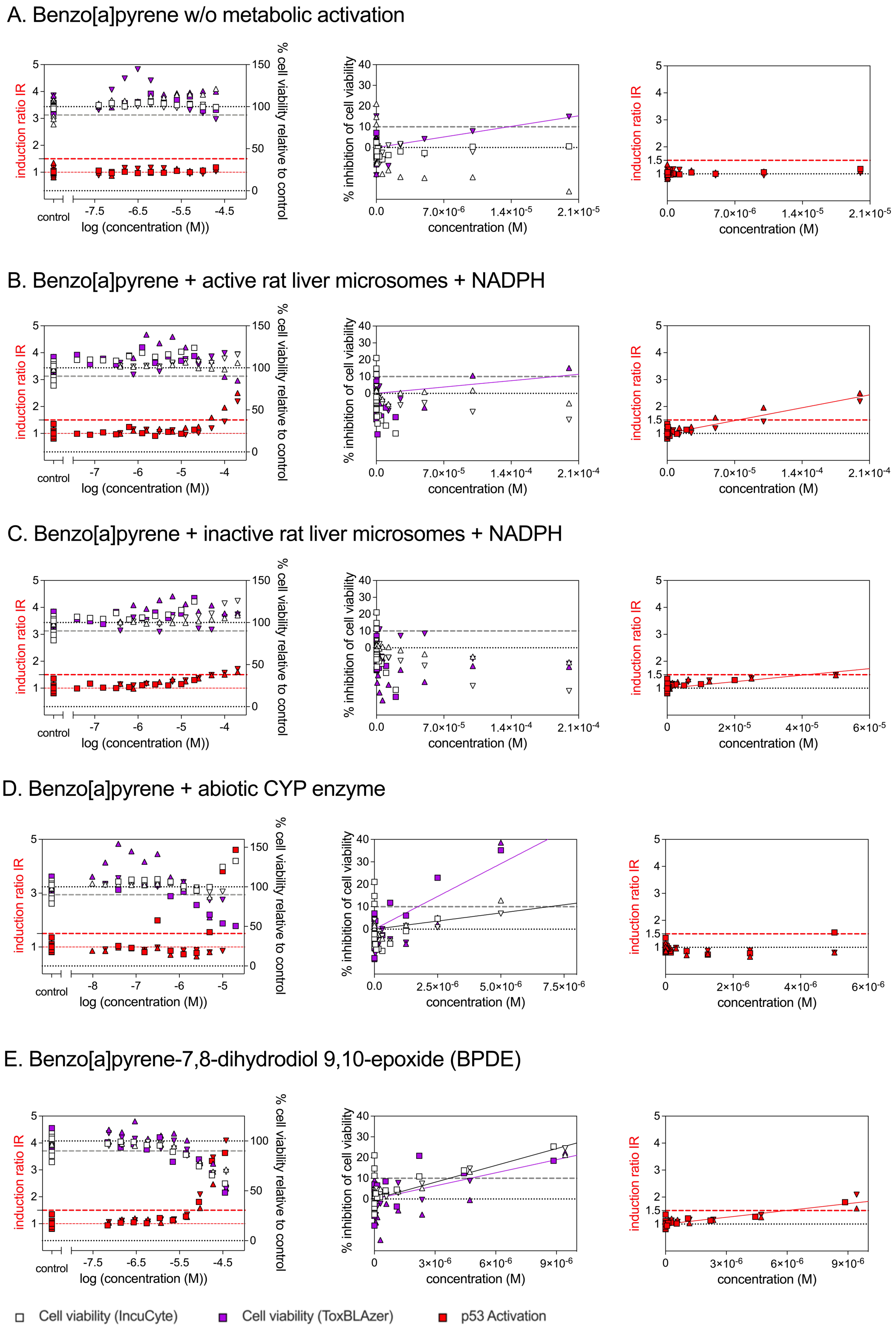

Figure S8. Concentration-response curves of benzo[a]pyrene (A) without metabolic activation, (B) incubated with active rat liver microsomes and NADPH, (C) incubated with inactive rat liver microsomes and NADPH, (D) activated with abiotic CYP enzyme and (E) concentration-response curves of benzo[a]pyrene-7,8-dihydrodiol 9,10-epoxide (BPDE). Different symbols indicate independent experimental replicates. Left plots: overview of all data on logarithmic scale, middle plots: linear fits of cytotoxicity, right plots: linear fit of p53 activation.

References

1. Neves, C. M. B.; Simoes, M. M. Q.; Domingues, F. M. J.; Neves, M.; Cavaleiro, J. A. S., BIOMIMETIC OXIDATION OF CARBAMAZEPINE WITH HYDROGEN PEROXIDE CATALYZED BY A MANGANESE PORPHYRIN. *Quimica Nova* **2012,** *35*, (7), 1477-U265.
